# 2-Hydroxy­amino-2-oxoacetohydrazide

**DOI:** 10.1107/S1600536810012341

**Published:** 2010-04-14

**Authors:** Oleksandra S. Trofymchuk, Svetlana V. Pavlova, Vladimir Bon, Alexander N. Boyko, Vasily Pekhnyo

**Affiliations:** a Department of Chemistry, National Taras Shevchenko University, Volodymyrska Str. 64, 01601 Kyiv, Ukraine; b Institute of General and Inorganic Chemistry, NAS Ukraine, prosp. Palladina 32/34, 03680 Kyiv, Ukraine

## Abstract

In the title compound, C_2_H_5_N_3_O_3_, the hydroxamic group adopts an *anti* orientation with respect to the hydrazide group. In the crystal, mol­ecules are connected by N—H⋯O and O—H⋯N hydrogen bonds into zigzag chains along the *c* axis.

## Related literature

For hydroxamic acids in biological chemistry, see: Kaczka *et al.* (1962 ▶); Komatsu *et al.* (2001 ▶). For the use of hydroxamic acids as strong chelating agents, see: Dobosz *et al.* (1999 ▶); Świątek-Kozłowska *et al.* (2000 ▶). For hydroxamic acids as the basis for the synthesis of metallacrowns compounds, see: Bodwin *et al.* (2001 ▶); Gumienna-Kontecka *et al.* (2007 ▶). For related structures, see: Sliva *et al.* (1997*a* ▶,*b* ▶); Mokhir *et al.* (2002 ▶); Fritsky *et al.* (2006 ▶); Moroz *et al.* (2008 ▶).
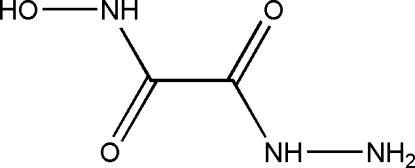


## Experimental

### 

#### Crystal data

C_2_H_5_N_3_O_3_
*M* *_r_* = 119.09
Monoclinic, 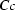

*a* = 9.3968 (7) Å
*b* = 3.6728 (2) Å
*c* = 12.7510 (8) Å
β = 95.598 (5)°
*V* = 437.97 (5) Å^3^
*Z* = 4
Mo *K*α radiation
μ = 0.17 mm^−1^
*T* = 77 K
0.12 × 0.10 × 0.07 mm

#### Data collection

Bruker APEXII diffractometer
Absorption correction: multi-scan (*SADABS*; Sheldrick, 2008 ▶) *T*_min_ = 0.980, *T*_max_ = 0.988
1149 measured reflections
445 independent reflections
404 reflections with *I* > 2σ(*I*)
*R*_int_ = 0.021

#### Refinement

*R*[*F* ^2^ > 2σ(*F* ^2^)] = 0.032
*wR*(*F* ^2^) = 0.077
*S* = 1.06
445 reflections
74 parameters
3 restraints
H-atom parameters constrained
Δρ_max_ = 0.19 e Å^−3^
Δρ_min_ = −0.20 e Å^−3^

### 

Data collection: *APEX2* (Bruker, 2007 ▶); cell refinement: *SAINT* (Bruker, 2007 ▶); data reduction: *SAINT*; program(s) used to solve structure: *SHELXS97* (Sheldrick, 2008 ▶); program(s) used to refine structure: *SHELXL97* (Sheldrick, 2008 ▶); molecular graphics: *ORTEP-3 for Windows* (Farrugia, 1999 ▶); software used to prepare material for publication: *SHELXL97*.

## Supplementary Material

Crystal structure: contains datablocks I, global. DOI: 10.1107/S1600536810012341/jh2142sup1.cif

Structure factors: contains datablocks I. DOI: 10.1107/S1600536810012341/jh2142Isup2.hkl

Additional supplementary materials:  crystallographic information; 3D view; checkCIF report

## Figures and Tables

**Table 1:** Hydrogen-bond geometry (Å, °)

*D*—H⋯*A*	*D*—H	H⋯*A*	*D*⋯*A*	*D*—H⋯*A*
N2—H1*N*2⋯O3^i^	0.88	2.02	2.813 (5)	149
O1—H1*O*1⋯N3^ii^	0.95	1.83	2.740 (4)	161
O1—H1*O*1⋯N3^ii^	0.95	1.83	2.740 (4)	161
N3—H1*N*3⋯O1^iii^	0.90	2.29	3.013 (3)	137
N3—H2*N*3⋯O1^iv^	0.93	2.44	3.024 (4)	121

Symmetry codes: (i) 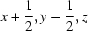; (ii) 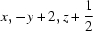; (iii) 
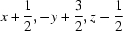
; (iv) 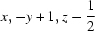.

**Fig. 1.**
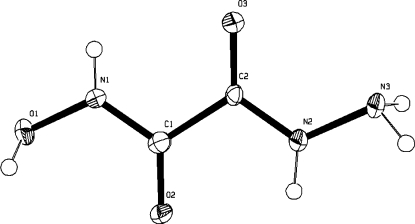
The molecular structure of (1), with 40% probability displacement ellipsoids showing the atom-numbering scheme employed.

**Fig. 2.**
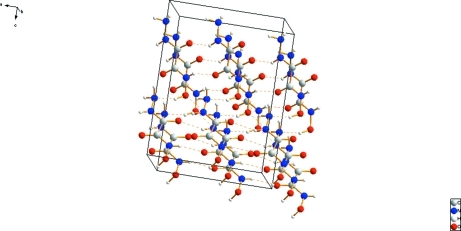
A packing diagram for (1) compound. Hydrogen bonds are indicated by dashed lines.

## supplementary crystallographic information

### Comment

Hydroxamic acids represent an important class of chelating agents and recently have been used for synthesis of metallocrown compounds (Dobosz *et al.*, 1999; Świątek-Kozłowska *et al.*, 2000; Bodwin *et al.*, 2001; Gumienna-Kontecka *et al.*, 2007). Besides, it is known that hydroxamic acids can act as inhibitors of enzymes as well as promising antitumor agents (Kaczka *et al.*,1962; Komatsu *et al.*, 2001). Therefore, study of new hydroxamic acids is timely and important research topic. As a part of our on-going work, we report the structure of the title compound (1), which comprises several groups capable to form hydrogen bond interactions.

The molecular structure of (1) is shown in Fig. 1. The hydroxamic group is in *anti-*position with respect to the hydrazide group. The carbonyl groups are in *trans*-position with respect to each other, and the NH_2_ group is *cis* with respect to the hydrazide carbonyl and the OH group is *cis* with respect to the hydroxamic carbonyl. The C1—N1 , N1—O1 , C1—O2, C2—O3, C2—N2, N2—N3 bond lengths are 1.319 (5) Å, 1.381 (5) Å, 1.242 (6) Å, 1.220 (5) Å, 1.321 (4) Å and 1.422 (6) Å respectively, adopt typical values to the hydroxamic and hydrazide groups (Sliva *et al.*, 1997a, b); Mokhir *et al.*, 2002; Fritsky *et al.*, 2006; Moroz *et al.*, 2008).

In the crystal the molecules are connected by N—H···O, O—H···N hydrogen bonds (Table 1, Fig. 2) into supramolecular zig-zag chains along the *c*-axis.

### Experimental

Compound (1) was synthesized as a white powder precipitate by addition of 1 equiv. of N_2_H_4_^. ^H_2_O to cooled ethanol solution of ethyl- 2-(hydroxyamino)-2-oxoacetate (250 mmol) following by recrystallization of the resulting product from water. Single crystals suitable for X-ray structure analysis were obtained by slow evaporation of aqueous solution at room temperature. ^1^H NMR (400 MHz, DMSO-*d_6_*, *δ*): 4.482 (s, 2H, NH_2_); 9.193 (br s, 1H, NH); 9.991(s, 1H, NH); 11.435 (br s, 1H, OH) ppm. ^13^C NMR (CDCl_3_, 100 MHz, *δ*): 162.07, 163.219 ppm.

### Refinement

The hydrogen atoms were located from the difference Fourier map and were constrained to ride on their parent atoms with *U*_ĩso_ = 1.2–1.5 *U*_eq_(parent atom). The highest peak is located 0.77 Å from atom C1 and the deepest hole is located 0.81 Å from atom N2. In the absence of significant anomalous scattering effects, 150 Friedel pairs were averaged in the final refinement.

### Crystal data

**Table:**

C_2_H_5_N_3_O_3_	*F*(000) = 248
*M**_r_* = 119.09	*D*_x_ = 1.806 Mg m^−^^3^
Monoclinic, *C**c*	Mo *K*α radiation, λ = 0.71073 Å
Hall symbol: C -2yc	Cell parameters from 1149 reflections
*a* = 9.3968 (7) Å	θ = 3.2–26.5°
*b* = 3.6728 (2) Å	µ = 0.17 mm^−^^1^
*c* = 12.7510 (8) Å	*T* = 77 K
β = 95.598 (5)°	Block, colourless
*V* = 437.97 (5) Å^3^	0.12 × 0.10 × 0.07 mm
*Z* = 4	

### Data collection

**Table:**

Bruker APEXII diffractometer	445 independent reflections
Radiation source: fine-focus sealed tube	404 reflections with *I* > 2σ(*I*)
horizontally mounted graphite crystal	*R*_int_ = 0.021
Detector resolution: 9 pixels mm^-1^	θ_max_ = 26.5°, θ_min_ = 3.2°
φ scans and ω scans with κ offset	*h* = −10→11
Absorption correction: multi-scan (*SADABS*; Sheldrick, 2008)	*k* = −4→4
*T*_min_ = 0.980, *T*_max_ = 0.988	*l* = −15→15
1149 measured reflections	

### Refinement

**Table:**

Refinement on *F*^2^	Primary atom site location: structure-invariant direct methods
Least-squares matrix: full	Secondary atom site location: difference Fourier map
*R*[*F*^2^ > 2σ(*F*^2^)] = 0.032	Hydrogen site location: inferred from neighbouring sites
*wR*(*F*^2^) = 0.077	H-atom parameters constrained
*S* = 1.06	*w* = 1/[σ^2^(*F*_o_^2^) + (0.0363*P*)^2^ + 0.3945*P*] where *P* = (*F*_o_^2^ + 2*F*_c_^2^)/3
445 reflections	(Δ/σ)_max_ < 0.001
74 parameters	Δρ_max_ = 0.19 e Å^−^^3^
3 restraints	Δρ_min_ = −0.20 e Å^−^^3^

### Special details

**Table:**

Geometry. All s.u.'s (except the s.u. in the dihedral angle between two l.s. planes) are estimated using the full covariance matrix. The cell s.u.'s are taken into account individually in the estimation of s.u.'s in distances, angles and torsion angles; correlations between s.u.'s in cell parameters are only used when they are defined by crystal symmetry. An approximate (isotropic) treatment of cell s.u.'s is used for estimating s.u.'s involving l.s. planes.
Refinement. Refinement of *F*^2^ against ALL reflections. The weighted *R*-factor wR and goodness of fit *S* are based on *F*^2^, conventional *R*-factors *R* are based on *F*, with *F* set to zero for negative *F*^2^. The threshold expression of *F*^2^ > 2σ(*F*^2^) is used only for calculating *R*-factors(gt) etc. and is not relevant to the choice of reflections for refinement. *R*-factors based on *F*^2^ are statistically about twice as large as those based on *F*, and *R*- factors based on ALL data will be even larger.

### Fractional atomic coordinates and isotropic or equivalent isotropic displacement parameters (Å^2^)

**Table:**

	*x*	*y*	*z*	*U*_iso_*/*U*_eq_	
C1	0.4570 (5)	0.6710 (10)	0.8492 (3)	0.0187 (10)	
C2	0.4385 (4)	0.8528 (9)	0.7408 (3)	0.0149 (9)	
N1	0.3439 (4)	0.7171 (8)	0.9018 (3)	0.0185 (8)	
H1N1	0.2695	0.8396	0.8732	0.022*	
N2	0.5546 (4)	0.8238 (9)	0.6907 (3)	0.0199 (8)	
H1N2	0.6295	0.7036	0.7194	0.024*	
N3	0.5578 (4)	0.9881 (9)	0.5899 (3)	0.0215 (9)	
H1N3	0.6479	1.0696	0.5880	0.032*	
H2N3	0.5534	0.8113	0.5371	0.032*	
O1	0.3428 (3)	0.5725 (8)	1.0016 (2)	0.0249 (8)	
H1O1	0.4139	0.6969	1.0456	0.037*	
O2	0.5674 (3)	0.5033 (7)	0.8820 (2)	0.0236 (8)	
O3	0.3288 (3)	1.0094 (7)	0.7077 (2)	0.0248 (9)	

### Atomic displacement parameters (Å^2^)

**Table:**

	*U*^11^	*U*^22^	*U*^33^	*U*^12^	*U*^13^	*U*^23^
C1	0.016 (3)	0.0199 (18)	0.020 (2)	0.0004 (15)	0.0007 (17)	−0.0040 (15)
C2	0.016 (2)	0.0162 (16)	0.013 (2)	0.0003 (14)	0.0037 (17)	−0.0026 (14)
N1	0.0142 (17)	0.0252 (17)	0.0162 (19)	0.0030 (13)	0.0019 (14)	0.0015 (14)
N2	0.0194 (19)	0.0256 (16)	0.015 (2)	0.0059 (14)	0.0050 (14)	0.0019 (14)
N3	0.020 (2)	0.0287 (16)	0.017 (2)	0.0034 (13)	0.0093 (15)	0.0020 (14)
O1	0.0266 (19)	0.0337 (15)	0.0150 (17)	−0.0005 (13)	0.0048 (13)	0.0048 (14)
O2	0.018 (2)	0.0294 (17)	0.024 (2)	0.0081 (12)	0.0057 (16)	0.0060 (12)
O3	0.020 (2)	0.0373 (18)	0.0173 (19)	0.0095 (12)	0.0025 (16)	0.0031 (12)

### Geometric parameters (Å, °)

**Table:**

C1—O2	1.243 (6)	N1—H1N1	0.8800
C1—N1	1.322 (4)	N2—N3	1.422 (6)
C1—C2	1.530 (4)	N2—H1N2	0.8800
C2—O3	1.220 (5)	N3—H1N3	0.9009
C2—N2	1.321 (4)	N3—H2N3	0.9332
N1—O1	1.380 (5)	O1—H1O1	0.9468
			
O2—C1—N1	125.4 (4)	O1—N1—H1N1	120.1
O2—C1—C2	122.5 (3)	C2—N2—N3	119.6 (4)
N1—C1—C2	112.1 (3)	C2—N2—H1N2	120.2
O3—C2—N2	125.5 (4)	N3—N2—H1N2	120.2
O3—C2—C1	122.4 (3)	N2—N3—H1N3	105.5
N2—C2—C1	112.1 (3)	N2—N3—H2N3	110.6
C1—N1—O1	119.8 (4)	H1N3—N3—H2N3	100.8
C1—N1—H1N1	120.1	N1—O1—H1O1	107.0
			
O2—C1—C2—O3	178.1 (5)	O2—C1—N1—O1	0.0 (6)
N1—C1—C2—O3	−2.3 (4)	C2—C1—N1—O1	−179.6 (3)
O2—C1—C2—N2	−3.1 (4)	O3—C2—N2—N3	0.8 (6)
N1—C1—C2—N2	176.5 (4)	C1—C2—N2—N3	−178.0 (3)

### Hydrogen-bond geometry (Å, °)

**Table:**

*D*—H···*A*	*D*—H	H···*A*	*D*···*A*	*D*—H···*A*
N2—H1N2···O3^i^	0.88	2.02	2.813 (5)	149
O1—H1O1···N3^ii^	0.95	1.83	2.740 (4)	161
O1—H1O1···N3^ii^	0.95	1.83	2.740 (4)	161
N3—H1N3···O1^iii^	0.90	2.29	3.013 (3)	137
N3—H2N3···O1^iv^	0.93	2.44	3.024 (4)	121

Symmetry codes: (i) *x*+1/2, *y*−1/2, *z*; (ii) *x*, −*y*+2, *z*+1/2; (iii) *x*+1/2, −*y*+3/2, *z*−1/2; (iv) *x*, −*y*+1, *z*−1/2.
